# G Protein-Mediated Allosteric Modulation of Ligand Binding in Class A GPCRs: Receptor–Ligand–Transducer Ensembles in Disease and Drug Discovery

**DOI:** 10.3390/ph19081312

**Published:** 2026-08-20

**Authors:** Yukiko Kurihara, Hiroki Kurihara

**Affiliations:** 1 Faculty of Nutrition Sciences, Japan Nutrition University, 3-9-21 Chiyoda, Sakado, Saitama 350-0288, Japan; 2 Isotope Science Center, The University of Tokyo, 2-11-16 Yayoi, Bunkyo-ku, Tokyo 113-0032, Japan; kuri-tky@umin.net; 3 Laboratory of Multicellular Dynamics, International Research Center for Medical Sciences, Kumamoto University, 2-2-1 Honjo, Chuo-ku, Kumamoto 860-0811, Japan

**Keywords:** GPCR, ternary complex, allostery, G protein, ligand binding, conformational ensemble, receptor activation

## Abstract

G protein-coupled receptors (GPCRs) are dynamic allosteric proteins whose signaling properties are governed by reciprocal communication between extracellular ligand-binding sites and intracellular transducer interfaces. Although classical pharmacological models established the concept that ligand binding and G protein coupling are thermodynamically linked, recent structural, biophysical, and computational studies have revealed a far more complex picture in which GPCRs exist as ensembles of interconverting conformational states. Accumulating evidence indicates that G proteins function not only as downstream signaling effectors but also as endogenous allosteric modulators. By reshaping receptor conformational landscapes, G protein coupling can influence the structure and dynamics of orthosteric ligand-binding pockets, thereby regulating ligand affinity, binding kinetics, and receptor selectivity. These findings support a bidirectional model of GPCR signaling in which information is transmitted not only from ligand-binding sites to intracellular signaling partners but also in the reverse direction through receptor-wide allosteric networks. Disease-associated mutations of endothelin A receptor (ETAR) provide in vivo evidence that structural perturbations located far from orthosteric ligand-binding sites can alter ligand recognition through long-range allosteric communication. In addition, emerging studies of positive allosteric modulators demonstrate the therapeutic potential of selectively stabilizing ligand–receptor–G protein complexes. These observations suggest that ligand recognition, receptor activation, and transducer coupling should be viewed as integrated properties of a dynamic receptor–ligand–transducer ensemble. This perspective provides a conceptual framework that links classical GPCR pharmacology, structural biology, disease mechanisms, and next-generation drug discovery.

## 1. Introduction

G protein-coupled receptors (GPCRs) constitute the largest family of membrane receptors in the human genome and regulate a remarkably broad range of physiological processes [1,2]. Their pharmacological importance is underscored by the fact that a substantial proportion of currently approved drugs exert their therapeutic effects through GPCR-mediated signaling pathways [3]. Despite their long-standing importance in physiology and pharmacology, our understanding of the molecular mechanisms underlying GPCR activation has undergone profound changes over the past several decades.

Early pharmacological studies generally viewed receptor activation as a relatively simple process in which agonist binding induces a signaling-competent receptor state that subsequently activates G proteins. In particular, the ternary complex (TC) model provided a unified explanation for high-affinity agonist binding and the role of receptor–G protein interactions, thereby establishing a conceptual foundation for modern GPCR pharmacology [4]. Subsequent discoveries of constitutive receptor activity led to the development of the extended ternary complex (ETC) model, while the cubic ternary complex (CTC) model further expanded this framework by incorporating the possibility of G protein interactions with inactive receptor conformations [5,6]. Collectively, these models shifted the perception of GPCRs from simple binary switches toward dynamic systems that populate multiple interconverting states.

At the time these pharmacological models were developed, the structural basis of receptor–G protein interactions remained largely speculative. Subsequently, advances in X-ray crystallography, nuclear magnetic resonance (NMR) spectroscopy, electron paramagnetic resonance (EPR), cryo-electron microscopy (cryo-EM), and molecular dynamics (MD) simulations have since provided unprecedented insights into the architecture and dynamics of GPCR–G protein complexes [1,7,8]. These studies have supported the view that GPCR activation cannot be adequately described as a transition between a single inactive state and a single active state. Rather, GPCRs exist as dynamic conformational ensembles whose distributions are continuously reshaped by ligands, transducers, and cellular context. Within this framework, G proteins should not be viewed solely as downstream signaling effectors.

Increasing evidence indicates that G protein coupling can also act in the reverse direction, altering receptor conformational equilibria and influencing the structure, dynamics, and accessibility of the orthosteric ligand-binding pocket [9]. This reciprocal communication challenges a strictly unidirectional view of GPCR signaling and supports a model in which extracellular ligand recognition and intracellular transducer coupling are linked through receptor-wide allosteric networks. Despite growing recognition of this phenomenon, important questions remain unresolved. It is still unclear how broadly G protein–dependent modulation of ligand binding applies across class A GPCR subtypes, how its molecular basis differs among receptor systems, and how such mechanisms contribute to disease pathogenesis and therapeutic intervention. An important example is provided by studies of endothelin A receptor (ETAR), in which disease-associated mutations located far from the orthosteric ligand-binding pocket alter ligand selectivity through long-range allosteric communication across the receptor [10].

In this review, we trace how classical pharmacological models of GPCR–G protein coupling have evolved into current ensemble-based views of receptor activation and allosteric regulation. We first summarize the conceptual framework provided by the ternary, extended ternary, and cubic ternary complex models, and then discuss structural and dynamic studies that have revealed how G protein coupling reshapes receptor conformational states. We next focus on representative class A GPCR systems to examine how transducer engagement can influence orthosteric ligand-binding pockets, ligand affinity, binding kinetics, and receptor selectivity. Finally, we consider how these mechanisms illuminate disease-associated alterations in ligand recognition and how stabilization of specific receptor–ligand–transducer states may be exploited for drug discovery.

The distinctive contribution of this review is not merely to revisit conformational ensembles, reverse allostery, or GPCR structural dynamics as individual concepts, but to place G protein-mediated regulation of ligand binding within the classical TC/CTC framework and to consider how this concept may be linked to disease-associated changes in ligand recognition and drug discovery. In particular, reverse allostery provides a useful perspective for understanding how mutations located far from the orthosteric ligand-binding pocket can influence ligand selectivity through long-range allosteric communication. By further connecting this disease-related perspective with recent examples of receptor–ligand–transducer ternary complex stabilization by allosteric modulators, this review emphasizes that GPCR pharmacology should be understood not only at the level of static orthosteric ligand binding, but also at the level of dynamic receptor–ligand–transducer ensembles.

## 2. From the Ternary Complex Model to the Cubic Ternary Complex Model

The conceptual basis for viewing GPCRs as dynamic allosteric systems emerged from classical pharmacological studies showing that agonist affinity is modulated by receptor–G protein interactions and guanine nucleotides.

### 2.1. Ternary Complex (TC) Model

Early studies of hormone receptor signaling already suggested that receptor activation is not determined solely by ligand binding. Rather, guanine nucleotides such as GTP, together with intracellular coupling factors later recognized as G proteins, were shown to modulate ligand affinity and receptor functional states [11,12,13]. These observations provided an important conceptual basis for viewing receptors as dynamic allosteric entities whose conformational equilibria are shaped by both extracellular ligands and intracellular transducers.

**Figure 1:**
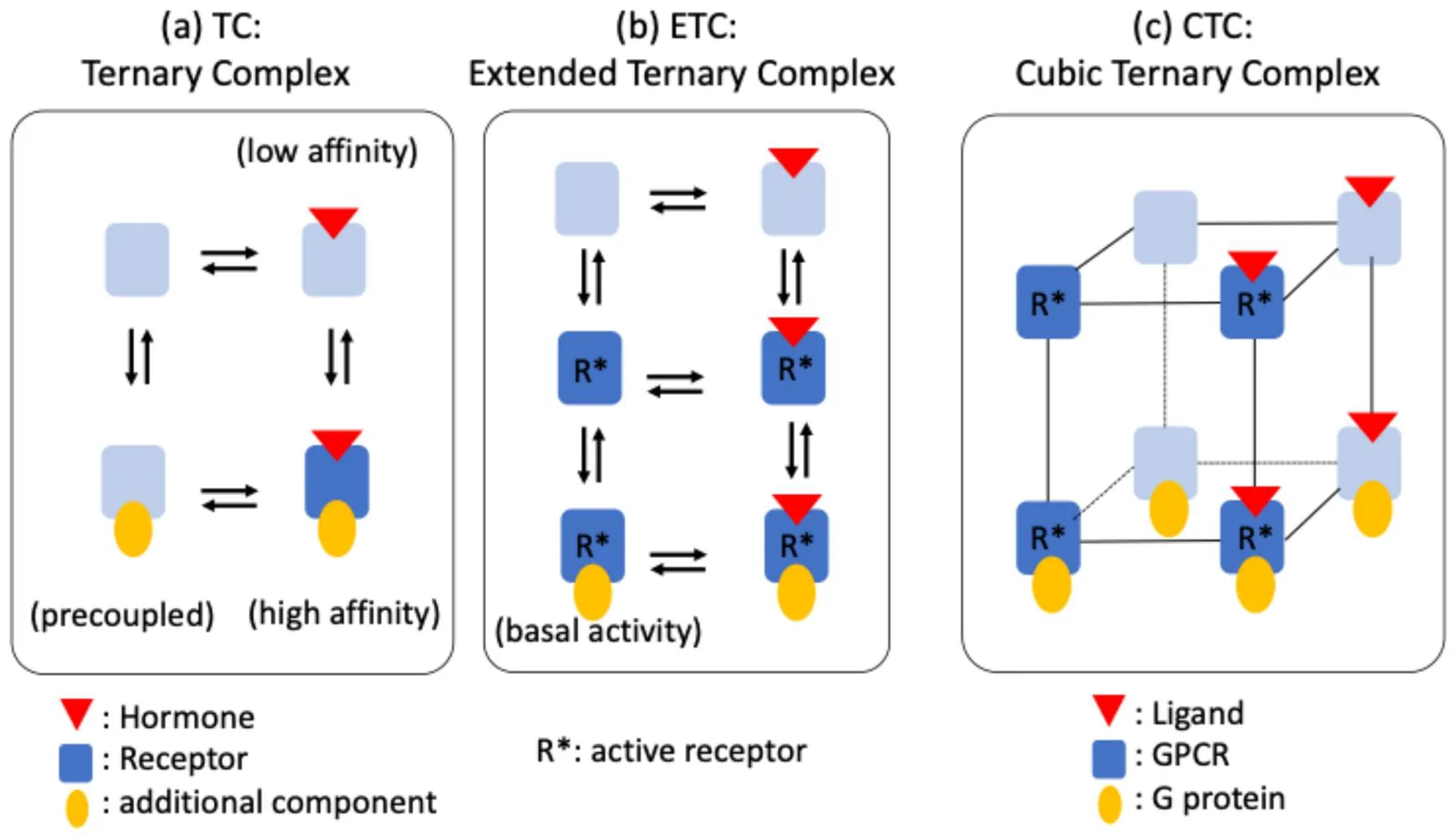
Classical pharmacological models of GPCR–G protein coupling. (**a**) TC model describes agonist-promoted formation of a high-affinity hormone–receptor–coupling component complex. (**b**) The ETC model incorporates spontaneous receptor activation and basal activity in the absence of ligand. (**c**) The CTC model allows ligand and G protein binding to both inactive and active receptor conformations.

Detailed pharmacological analyses of the β-adrenergic receptor in frog erythrocytes demonstrated that agonist binding occurs in at least two affinity states, whose relative proportions and affinities are determined by both agonist intrinsic activity and the presence of guanine nucleotides. To explain these observations, the classical TC model was introduced, in which a hormone or agonist (H), a receptor (R), and an additional membrane component (X) form a high-affinity complex (HRX) [4] (Figure 1a). In this model, agonists not only bind to the receptor but also promote interactions between the receptor and component X. Agonist intrinsic activity correlates with the affinity of X for the HR complex, whereas guanine nucleotides act primarily by destabilizing the high-affinity HRX complex.

Importantly, this model introduced the concept that agonist affinity is not determined solely by the receptor itself but is strongly influenced by intracellular components interacting with the receptor—factors later identified as G proteins. However, receptor activation was assumed to depend primarily on agonist-induced formation of the ternary complex, and the model therefore could not adequately account for receptor activity in the absence of ligands, namely constitutive activity.

### 2.2. Extended Ternary Complex (ETC) Model

To overcome this limitation, the ETC model was proposed. Studies of constitutively active β2-adrenergic receptor mutants revealed that the classical TC model could not sufficiently explain ligand-independent receptor activation, increased agonist affinity, or enhanced intrinsic activity of partial agonists. The ETC model explicitly incorporated spontaneous isomerization of the receptor from an inactive state (R) to an active state (R*) even in the absence of ligand (Figure 1b).

In this framework, the receptor exists in equilibrium between inactive (R) and active (R*) conformations, and agonists shift this equilibrium toward the active state. A key feature of the model is that R* can exist without ligand binding; consequently, formation of an R*G complex with a G protein generates basal signaling activity, providing a mechanistic explanation for constitutive activity [5,14,15,16].

### 2.3. Cubic Ternary Complex (CTC) Model

Despite its advances, the ETC model assumes that G proteins interact primarily with the active receptor state (R*) and does not explicitly allow G protein binding to inactive receptors. From a thermodynamic perspective, however, inactive receptors should also be capable of interacting with G proteins, regardless of whether a ligand is bound. This concept was incorporated into the CTC model (Figure 1c). The CTC model generalizes the ETC model by allowing G proteins to bind both inactive and active receptor conformations. This addition enables a more complete equilibrium description of the interactions among ligands, receptors, and G proteins. In its simplest form, involving one receptor species, one G protein species, and one ligand, the model is represented by eight receptor states located at the vertices of a cube [6]. These eight states correspond to all possible combinations of ligand-bound versus ligand-free, G protein-bound versus G protein-free, and inactive versus active receptor conformations. Conceptually, they can be represented as R, R*, AR, AR*, RG, R*G, ARG, and AR*G. Here, R and AR denote inactive receptor states, whereas R* and AR* represent active receptor states, with additional distinctions based on G protein association. This framework highlights that G proteins are not merely downstream signaling partners but also allosteric modulators capable of binding inactive receptor conformations and thereby altering the equilibrium distribution of receptor states.

The CTC model also provides a useful framework for understanding inverse agonism. Rather than simply competing with agonists for receptor binding, inverse agonists shift the receptor population toward inactive conformations that contribute less to basal signaling. Consistent with this concept, studies of the histamine H2 receptor demonstrated that the inverse agonist tiotidine biases the receptor population toward a signaling-incompetent G protein-coupled state, likely corresponding to inactive receptor–Gs complexes, thereby reducing the availability of Gs proteins for productive signaling. These findings are consistent with interpretations based on the CTC model [17]. Within this framework, the CTC model offers a different way to view ligand affinity and efficacy. Rather than treating affinity as an intrinsic constant of a ligand for a receptor, this model considers the receptor as a population of interconverting conformational states. A ligand may bind these states with different affinities, and the experimentally observed affinity reflects which states are preferentially populated under a given condition. Because G proteins and other intracellular partners can alter this population, they can also change the apparent affinity of the ligand. The same principle applies to efficacy. A ligand is effective not simply because it binds the receptor, but because it stabilizes receptor conformations that can productively couple to downstream signaling proteins. In this way, the CTC model links ligand binding, receptor conformation, and signaling output within a single ensemble-based framework [6].

Together, the TC, ETC, and CTC models established the pharmacological basis for viewing GPCRs as dynamic ensembles in which ligand binding, receptor activation, and G protein coupling are mutually coupled. This concept provides the foundation for the later structural and biophysical understanding of G protein-mediated allosteric regulation of ligand binding.

## 3. GPCR–G Protein Coupling: From Pharmacological Models to Structural and Dynamic Understanding

The pharmacological models discussed above predicted receptor–ligand–G protein complexes but did not define their structural organization or dynamic transitions. These questions have been addressed increasingly by recent structural and biophysical studies, which have revealed the molecular basis of GPCR–G protein coupling and nucleotide release (Table 1).

### 3.1. Structural Understanding of GPCR–G Protein Complexes

**Figure 2:**
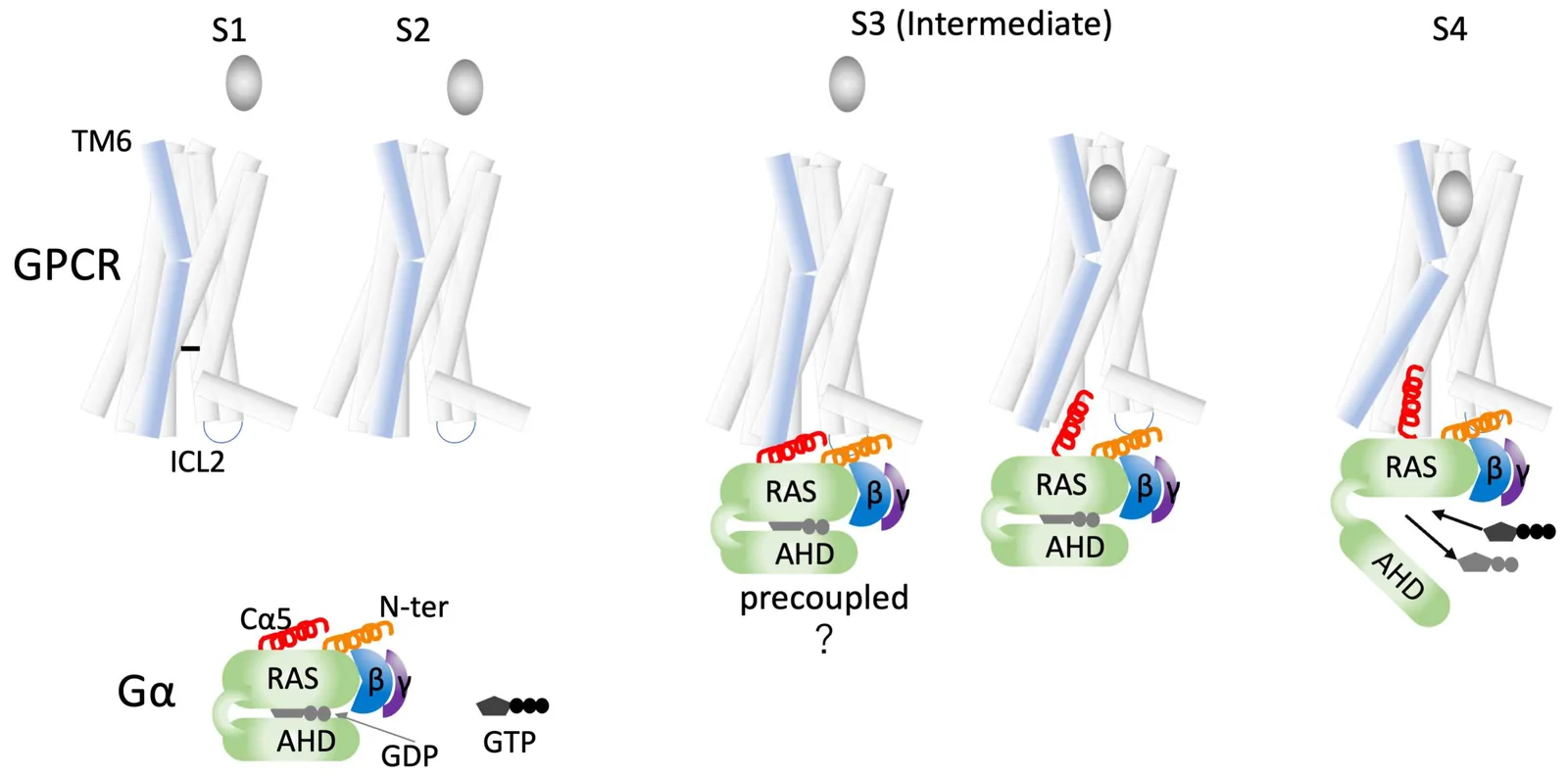
Structural and dynamic intermediates during GPCR–G protein complex formation. GPCR–G protein coupling proceeds through a series of transient conformational states before formation of the stable nucleotide-free complex. Early interactions with GDP-bound G proteins, including engagement of the Gα C-terminal α5 helix, can promote GDP release and contribute to receptor–G protein selectivity. The question mark denotes putative or incompletely defined transient intermediate states during GPCR–G protein complex formation. RAS; Ras-like GTPase domain, AHD; α-helical domain, Cα5; C-terminal region of the α5 helix of Gα, N-ter; N-terminal region of Gα.

A major breakthrough was the determination of the crystal structure of the β2-adrenergic receptor (β2AR)–Gs complex. This structure provided the first high-resolution visualization of an active-state ternary complex composed of agonist-bound β2AR and nucleotide-free heterotrimeric Gs, representing the first atomic-level view of GPCR-mediated transmembrane signal transduction. The structure revealed an outward displacement of approximately 14 Å in the cytoplasmic end of TM6 and an extension of the cytoplasmic segment of TM5, structural features that are now recognized as hallmarks of a receptor conformation stabilized cooperatively by agonist binding and G protein engagement. On the G protein side, the α-helical domain (AHD) of Gαs was observed to undergo a substantial separation from the Ras-like GTPase domain [7]. Furthermore, hydrogen–deuterium exchange mass spectrometry (HDX-MS) demonstrated that binding of agonist-occupied β2AR to GDP-bound Gs induces dynamic changes in the N-terminus and C-terminal region of the α5 helix of Gαs, destabilizing the GDP-binding pocket and facilitating GDP release [18] (Figure 2).

An important concept that emerged from the β2AR–Gs structure is that G protein coupling is not merely a simple binding event. Rather, it represents a reciprocal allosteric process that induces extensive conformational changes in both the receptor and the G protein. On the receptor side, outward movement of TM6 creates a cytoplasmic cavity capable of accommodating the C-terminal α5 helix of Gα. On the G protein side, receptor engagement is propagated to the nucleotide-binding pocket, promoting GDP dissociation. GPCR–G protein coupling can therefore be viewed as a continuous allosteric signaling process, transmitting information from the orthosteric ligand-binding site to the cytoplasmic G protein interface and ultimately to the nucleotide-binding pocket within the G protein itself [19].

Following the determination of the β2AR–Gs structure, the crystal structure of the adenosine A2A receptor (A2AR) bound to engineered mini-Gs further demonstrated both the general principles and receptor-specific features of G protein coupling in class A GPCRs. In the A2AR–mini-Gs complex, mini-Gs engages the receptor through an extensive interaction interface, and the transition from the agonist-bound active-intermediate state to the G protein-bound active state is accompanied by an approximately 14 Å outward displacement of the cytoplasmic end of TM6 [20].

Such structural analyses of GPCR–G protein complexes have contributed not only to our understanding of receptor activation mechanisms, but also to elucidating the molecular basis of G protein selectivity. For example, the cryo-EM structure of the active-state 5-HT2A serotonin receptor (HTR2A)–Gq complex bound to the hallucinogen 25CN-NBOH revealed structural determinants that govern HTR2A–Gq interactions [21]. These findings highlight the importance of both cytoplasmic rearrangements on the receptor side and the interaction interface on the G protein side, particularly the C-terminal α5 helix of Gq, in defining coupling selectivity toward specific G protein families.

### 3.2. Conformational Dynamics Revealed by NMR, EPR, and Nanobodies

In addition to these static structural analyses, biophysical approaches such as NMR and EPR have demonstrated that GPCRs are not single, static structures, but rather dynamic molecules that exist in equilibrium among multiple conformational states. For example, a study using site-specific ^19^F-NMR labeling of β2AR showed that the cytoplasmic regions of TM6 and TM7 adopt distinct ligand-dependent conformational states. Agonists primarily shift TM6 toward a state associated with G protein activation, whereas β-arrestin-biased ligands have been reported to preferentially influence the conformational state of TM7 [22].

Furthermore, analyses of intracellular signaling and conformational changes using methods such as FRET (fluorescence resonance energy transfer) and BRET (bioluminescence resonance energy transfer) support the idea that ligand-dependent receptor conformational changes and phosphorylation patterns at the receptor C terminus, namely the phosphorylation barcode, are involved in determining the mode of arrestin engagement and the selectivity of downstream signaling. These findings have provided an important basis for understanding biased signaling not as the consequence of a single active structure, but as a structural ensemble shaped by the interplay among ligands, receptors, G proteins, and arrestins [8,23,24].

**Figure 3:**
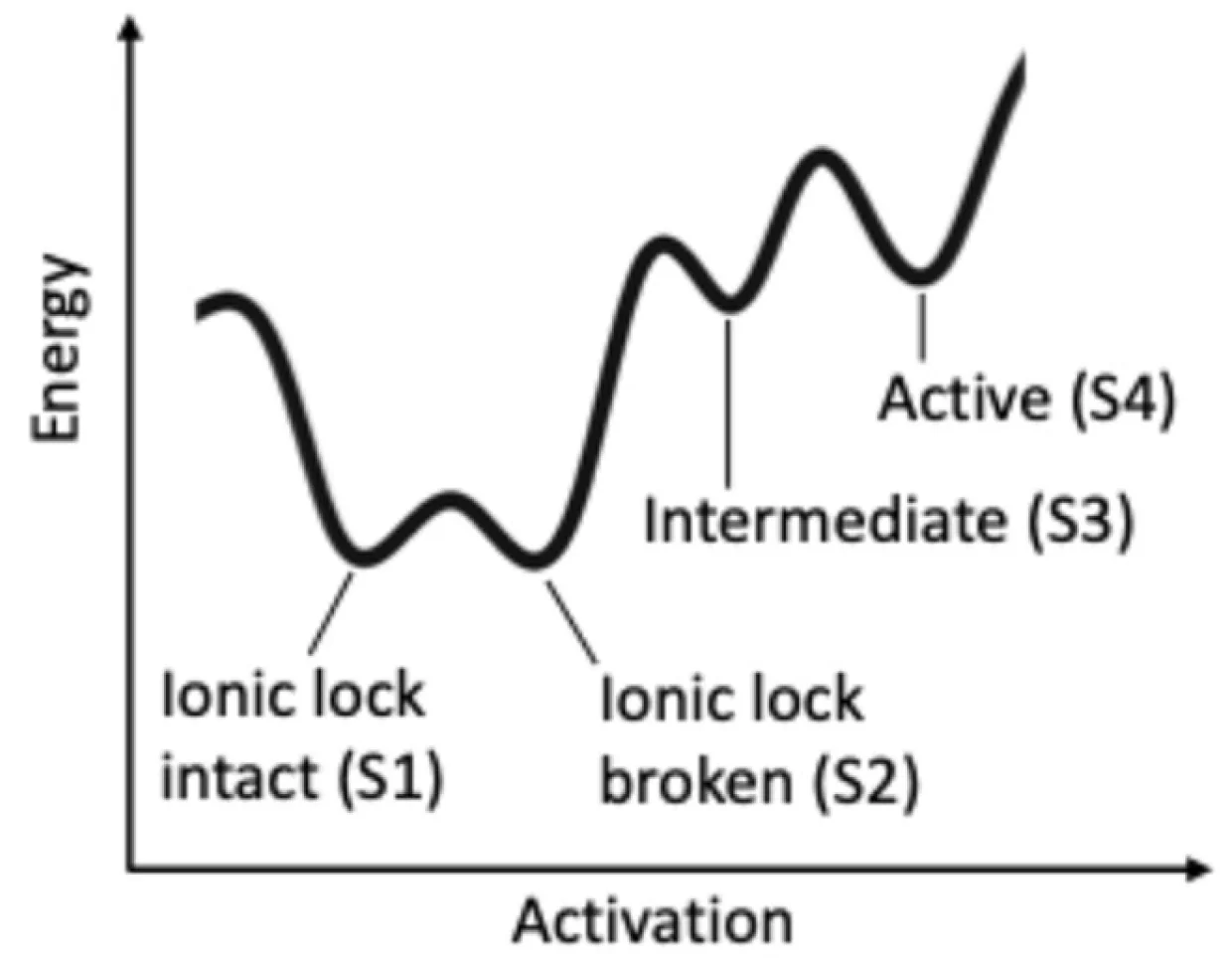
Schematic representation of the β2AR conformational energy landscape. Ligand-free β2AR predominantly samples inactive conformations (S1, S2), whereas agonist binding shifts the ensemble toward active-like states (S4). Full stabilization of the active state requires cooperative engagement of an agonist and a G protein or G protein–mimetic nanobody. Adapted from Manglik et al. [25], with permission.

Combined analyses using ^19^F-NMR, EPR, and DEER (double electron–electron resonance) spectroscopy further characterized cytoplasmic conformational dynamics of β2AR. In ligand-free and inverse agonist-bound states, the receptor predominantly exchanges between two inactive conformations, designated S1 and S2. S1 corresponds to an inactive state containing the ionic lock formed between R131^3.50^ in TM3 and E268^6.30^ in TM6, whereas S2 represents an inactive state in which this ionic lock is disrupted. Although agonists shift the equilibrium toward conformations capable of coupling to G proteins, agonist binding alone is insufficient to fully stabilize the active receptor state. Formation of the fully active conformation (S4) requires cooperative stabilization by both an agonist and either a G protein or a G protein–mimetic nanobody such as Nb80 [25,26] (Figure 3).

These observations support the concept that GPCR activation involves redistribution among multiple conformational states rather than conversion between two discrete inactive and active structures [1,8,27].

### 3.3. Intermediate States During GPCR–G Protein Complex Formation and G Protein Selectivity

However, these structures alone do not fully explain how receptors encounter GDP-bound G proteins, at which stage coupling specificity is determined, or how the complex proceeds toward the nucleotide-free state. The nucleotide-free GPCR–G protein complexes observed by crystallography and cryo-EM represent experimentally stabilized states and are thought to exist in living cells only as transient reaction intermediates or short-lived states. Time-resolved structural mass spectrometry approaches, including HDX-MS and hydroxyl radical-mediated protein footprinting with mass spectrometry (HRF-MS), have suggested that one or more transient intermediate states exist before the formation of the final nucleotide-free complex. These intermediates are not merely short-lived structures along the reaction pathway but may function as selectivity filters that determine G protein coupling specificity.

After ligand-bound β2AR is mixed with Gs, GDP release is completed within 10 s, whereas the structural changes corresponding to the stable nucleotide-free complex observed in crystal and cryo-EM structures occur more slowly. This suggests that GDP release is triggered by an early intermediate that precedes formation of the final stable complex [28]. In addition, in the early β2AR–Gs^GDP^ intermediate, the C-terminal α5 helix of Gαs binds to β2AR in an orientation distinct from that observed in the final nucleotide-free complex. The Gs-specific barcode residues R389^Gαs^ and E392^Gαs^ may participate in receptor recognition through interactions with residues surrounding the DRY motif of β2AR, including R131^3.50^, Y141^ICL2^, and T68^2.39^. Because these residues are exposed on the surface of GDP-bound Gs and are required for efficient β2AR–Gs coupling, this interaction in the early intermediate may provide a molecular basis for G protein subtype recognition before formation of the stable nucleotide-free complex [29,30]. Recent structural snapshots of the μ-opioid receptor (μOR) further support this stepwise view by showing that agonist efficacy correlates with reduced GDP affinity and enhanced nucleotide exchange in the G protein, whereas antagonists increase GDP affinity and thereby suppress receptor activation [31]. This principle is not limited to Gs-coupled receptors. In the cryo-EM structure of the Gq-coupled 5-HT2A serotonin receptor bound to the hallucinogen 25CN-NBOH, receptor activation is accompanied by cytoplasmic rearrangements, including outward movement of TM5 and TM6, inward displacement of TM7, ordering of ICL2, disruption of the E/DRY ionic lock, and rearrangement of W336^6.48^ and the P-I-F motif. These changes generate an intracellular interface that accommodates Gq, with the C-terminal α5 helix contributing to the receptor–G protein contact surface. Thus, the HTR2A–Gq structure extends the general importance of the Gα C-terminal region from Gs-coupled receptor complexes to a Gq-coupled receptor context [21]. Together, these studies suggest that GPCR–G protein selectivity cannot be fully understood from the final nucleotide-free complex alone. Instead, subtype-selective coupling may be shaped through a stepwise process, in which early interactions with GDP-bound G proteins and subsequent stabilization of the nucleotide-free complex both contribute to the formation of the final receptor–G protein interface (Figure 2).

Receptor conformational dynamics provide an additional layer to this stepwise view of GPCR activation and coupling. In β1AR, agonist binding establishes an equilibrium among two inactive states, S1 and S2, and a pre-active form, with the population of the pre-active form increasing as ligand efficacy increases. Coupling to a Gs-mimetic nanobody further promotes formation of a fully active ternary receptor complex, the abundance of which correlates with agonist efficacy. These observations suggest that a pre-existing conformational ensemble of the receptor provides a structural basis for ligand efficacy [32]. Pressure-resolved DEER spectroscopy has further revealed rare conformational states of β2AR, showing that even ligand-free β2AR contains a minor population with the outward TM6 displacement characteristic of active-like conformations. This population exists in equilibrium with inactive conformations and provides structural support for conformational selection as a basis for basal activity [33]. Finally, NMR spectroscopy and MD simulations of β2AR coupling to Gs and Gi1 showed that, although many receptor conformational changes are shared between the two complexes, distinct differences occur at the G protein-binding interface, particularly at ICL2. These findings indicate that both coupling preference and coupling promiscuity should be understood in terms of a dynamic receptor–G protein interface, rather than as properties encoded only in a fixed final complex [34] (Figure 2).

### 3.4. G Protein–First Model

The process of GPCR–G protein complex formation has traditionally been viewed through a ligand-first mechanism, in which an agonist first binds to an inactive GPCR, shifts the receptor toward an active-like conformation, and subsequently promotes G protein coupling. However, accumulating structural and biophysical evidence suggests that agonist binding alone is often insufficient to fully stabilize the active receptor conformation and that interactions with a G protein or a G protein–mimetic partner are required to achieve complete activation. These findings have raised the possibility that GPCRs and G proteins may form preassociated or precoupled complexes before agonist binding occurs.

FRET and BRET-based studies suggested that some GPCRs can exist in preassembled complexes with heterotrimeric G proteins in living cells, and agonist binding can induce conformational rearrangements within such preexisting complexes rather than simple association of freely diffusing components [35,36]. In particular, M3 muscarinic receptors were shown to form inactive-state complexes with Gq heterotrimers through a C-terminal polybasic motif, and disruption of this motif impaired preassembly and slowed Gq activation [37]. Similarly, 5-HT7 receptors were reported to associate with Gs heterotrimers in an inactive-state complex that contributes to the dynamic range of agonist-induced signaling [38]. These studies provide experimental support for the existence of receptor–G protein complexes before agonist binding in selected receptor systems.

More recently, molecular dynamics and metadynamics simulations across 15 class A GPCRs—including opioid, adrenergic, adenosine, chemokine, muscarinic, cannabinoid, serotonin, and dopamine receptors—led to the proposal of a G protein–first (GP-first) mechanism [39]. In this model, inactive G proteins can interact directly with inactive GPCRs before agonist binding, promoting opening of tight intracellular TM3–TM6 interactions and partial insertion of the Gα C-terminal α5 helix into the receptor cytoplasmic cavity, thereby forming a precoupled GPCR–G protein complex (Figure 2). This framework provides an alternative perspective on GPCR–G protein coupling. Rather than viewing coupling solely as an agonist-dependent recruitment process, it suggests that receptor–G protein interactions may exist prior to ligand binding and that agonists subsequently drive activation of an already associated complex. In particular, the idea that a G protein can partially open the intracellular face of an inactive receptor and promote insertion of the α5 helix is conceptually consistent with the inactive receptor–G protein complex (RG) postulated in the cubic ternary complex model.

Nevertheless, these findings do not establish that a GP-first pathway is universal or dominant for class A GPCR activation. Other live-cell studies support rapid collision coupling and failed to detect significant precoupling for α2A-adrenergic receptors [40] and several Gi-coupled receptors under the conditions examined [41]. In addition, approaches based on FRET, BRET, FRAP, or two-photon polarization microscopy are sensitive to probe position, protein expression level, membrane organization, and basal receptor activity, and therefore may report molecular proximity or basal coupling rather than a stable inactive receptor–G protein complex. Thus, the available experimental evidence supports the possibility of GPCR–G protein preassociation in some systems, but also indicates that its prevalence, lifetime, structural form, and physiological contribution are likely receptor-, G protein-, and cellular context-dependent.

The GP-first model is best regarded as one possible activation route within a broader ensemble-based framework. Ligand-first recruitment, inactive-state preassembly, basal coupling, and stepwise intermediate formation may coexist, and their relative contributions may vary among receptor–transducer pairs. Future studies combining time-resolved structural methods, single-molecule approaches, ligand-binding kinetics, and functional assays under near-physiological expression levels will be required to determine when preassociated receptor–G protein complexes represent productive intermediates in signaling rather than nonproductive or regulatory assemblies.

Taken together, these findings support an ensemble-based view in which GPCR–G protein coupling is not merely a terminal event after ligand binding, but one component of a dynamic network of inactive, preassociated, intermediate, and fully active states. This framework provides the basis for understanding how G protein engagement can reshape receptor conformational ensembles and influence ligand recognition, affinity, and kinetics, as discussed in the following section.

Structural, spectroscopic, computational, and live-cell studies have refined the classical pharmacological view of GPCR–G protein coupling. Together, these studies indicate that coupling is not simply a terminal event after ligand binding, but involves dynamic transitions among inactive, preassociated, intermediate, and fully active receptor–transducer states.

**Table 1:** Representative advances in GPCR–G protein coupling.

Conceptual Topic	System	Approach	Key Finding	Implication	Ref.
Active TC structure	β2AR-Gs	X-ray crystallography, HDX-MS	Outward TM6 movement, Gαs α5-helix insertion, and opening of the Gαs α-helical domain.	Established an atomic framework for transmembrane signaling and receptor-catalyzed nucleotide release.	[7,18]
Reciprocal receptor-GP conformational coupling	β2AR-Gs	Single- molecule FRET	Gs coupling stabilizes active β2AR conformations, while receptor conformational states reciprocally regulate G protein activation.	Demonstrated bidirectional allosteric communication between receptor and G protein, supporting an ensemble-based view of receptor–ligand–transducer coupling.	[19]
Receptor- specific Gs coupling	A2AR-mini-Gs	X-ray crystallography	Active-state stabilization with large outward TM6 movement but limited extracellular pocket rearrangement.	Showed that intracellular activation and extracellular pocket remodeling can be receptor dependent.	[20]
Gq-coupling interface	5-HT2A-Gq	Cryo-EM	Cytoplasmic rearrangements and Gq α5-helix interactions define the coupling interface.	Linked G protein selectivity to receptor-specific intracellular contacts and conserved activation motifs.	[21]
Ligand- dependent conformational ensembles	β2AR	^19^F-NMR, EPR, DEER, nanobody stabilization	Multiple inactive and active-like states; full activation requires agonist plus G protein or nanobody.	Supported cooperative stabilization of active states by ligands and transducers.	[22,25,26]
Arrestin barcode and biased signaling	β2AR–β- arrestin	Phospho-mapping, assays, ^19^F-NMR	Distinct β2AR phosphorylation patterns encode differential β-arrestin functions.	Provided original experimental support for the GPCR phosphorylation barcode concept.	[23,24]
Stepwise complex formation	β2AR-Gs	Time- resolved HDX-MS, HRF-MS	Rapid GDP release occurs through early intermediates before the stable nucleotide-free complex forms.	Identified transient intermediates as key contributors to activation and coupling specificity.	[28,29]
Dynamic coupling interface	β2AR-Gs, β2AR-Gi	FRET, MD, solution NMR	The Gα α5 helix and ICL2 shape shared and G protein-specific coupling features.	Suggested that coupling preference and promiscuity arise from a dynamic receptor–G protein interface.	[30,34]
Ligand efficacy conformational selection	β1AR	^19^F-NMR	Ligands with different efficacies differentially populate inactive, pre-active, and fully active states.	Linked ligand efficacy to shifts in preexisting conformational equilibria.	[32]
Rare active-like states	β2AR	Pressure- resolved DEER	Ligand-free receptors contain a minor active-like population with outward TM6 displacement.	Supported conformational selection as a basis for basal activity and coupling competence.	[33]
Preassociated complexes	M3R-Gq, 5-HT7-Gs	FRET, BRET, live-cell imaging	Some receptors form inactive-state or preassembled complexes before agonist stimulation.	Suggested that preassociation can contribute to signaling in selected receptor systems.	[35,36,37,38]

## 4. Reverse Allostery from G Proteins to Ligand-Binding Pockets: Closed Active States and Beyond

Bidirectional allosteric communication between ligand-binding and transducer-binding sites has been extensively discussed from both computational and structural perspectives [42].

### 4.1. The Concept of the Closed Active State

As discussed in the previous section, formation of a complex between a ligand-bound GPCR and a G protein involves opening of the receptor’s intracellular cavity, insertion of the C-terminal α5 helix of the Gα subunit, and reorganization of the nucleotide-binding pocket within Gα. However, these structural changes are not confined to the intracellular side of the receptor. Conformational rearrangements induced or stabilized by G protein coupling can be transmitted through the seven-transmembrane helical bundle to the extracellular side, thereby influencing the architecture of the orthosteric ligand-binding pocket, ligand association and dissociation kinetics, and ultimately apparent ligand affinity.

One of the clearest demonstrations of this “reverse allostery” from the G protein to the ligand-binding pocket was provided by DeVree, Sunahara, Kobilka, and colleagues. Using the β2AR–Gs system, they showed through pharmacological and biochemical analyses that binding of nucleotide-free Gs stabilizes a “closed” active conformation of β2AR in which access to the ligand-binding pocket is restricted. Importantly, this phenomenon was observed not only in agonist-induced high-affinity states but also in β2AR–nucleotide-free Gs complexes formed through basal receptor activity. In this state, G protein binding inhibits the association of agonists, partial agonists, antagonists, and inverse agonists, while simultaneously slowing the dissociation of already bound ligands [9]. Thus, G proteins should not be viewed merely as downstream signaling partners that enhance agonist affinity; rather, they function as endogenous allosteric modulators that regulate access to the orthosteric binding pocket and control ligand residence time.

This concept provides a structural explanation for the classical pharmacological observation that G protein coupling stabilizes high-affinity agonist binding. More broadly, it suggests that GPCR activation should not be regarded as a strictly unidirectional process from ligand to G protein, but rather as a bidirectional allosteric network in which information can also flow from the intracellular G protein interface back to the extracellular ligand-binding site.

### 4.2. How Does G Protein Coupling Alter the Ligand-Binding Pocket?

#### 4.2.1. β2-Adrenergic Receptor (β2AR)

**Figure 4:**
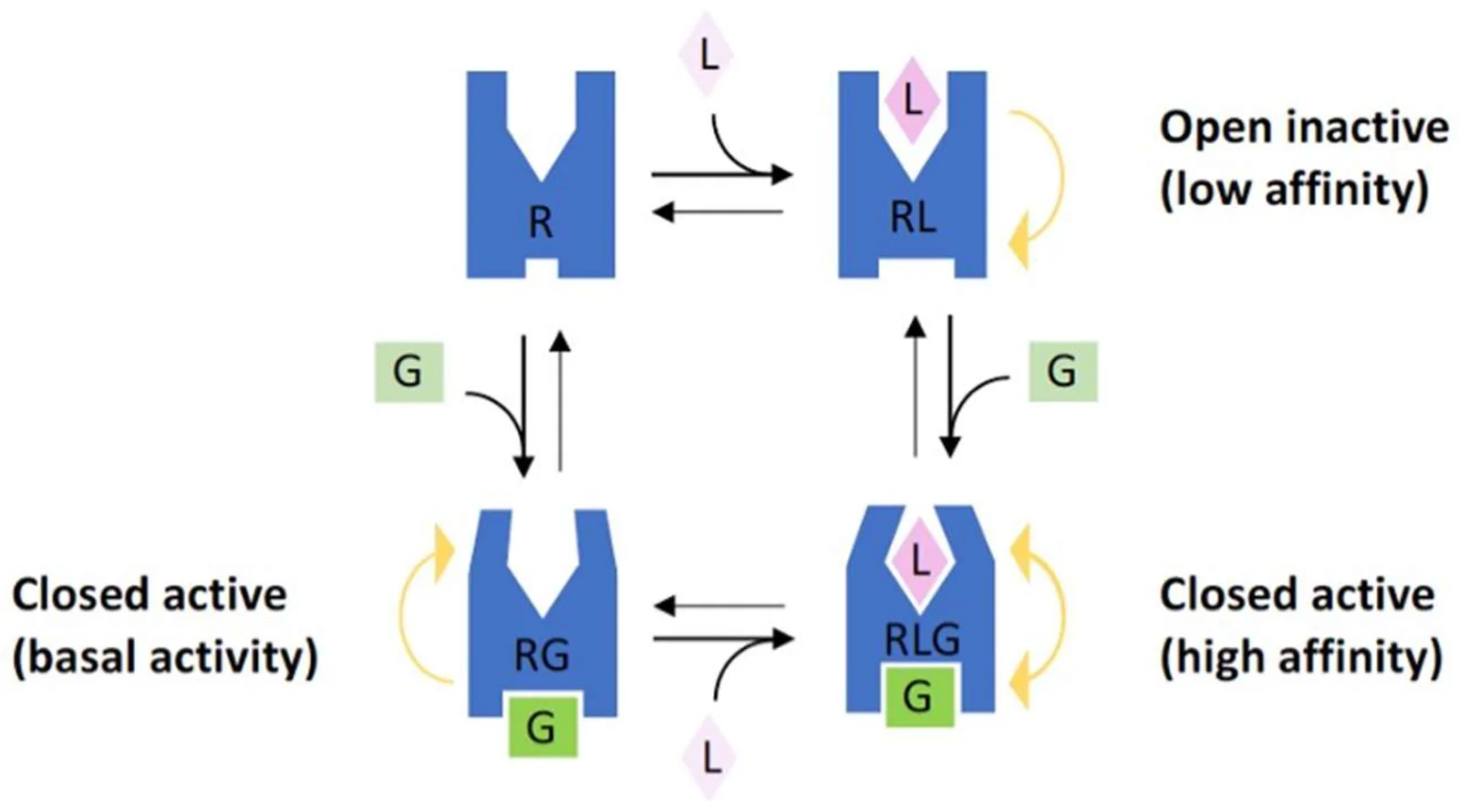
G protein-mediated reverse allostery toward the ligand-binding pocket. G protein coupling at the intracellular surface can be transmitted through the transmembrane helical bundle to the extracellular ligand-binding pocket, thereby altering pocket accessibility, ligand-binding kinetics, and apparent ligand affinity. Reproduced from Supplemental Figure 9 in Kurihara et al. [10], licensed under CC BY 4.0.

The β2AR is the prototypical receptor in which the concept of a G protein–induced closed active state has been most clearly established. Upon activation, outward displacement of TM6 on the intracellular side creates a binding cavity for the G protein, whereas on the extracellular side, ECL2 and the upper portion of TM7 undergo conformational rearrangements that promote closure of the entrance to the orthosteric ligand-binding pocket. In particular, the close positioning of Phe193^ECL2^ in ECL2 and Tyr308^7.35^ in TM7 forms a “lid-like” structure that covers the extracellular opening of the binding pocket and is regarded as a hallmark of the closed active state. Lys305^7.32^ may further stabilize this lid-like arrangement through altered interactions with Asp192^ECL2^ and the backbone carbonyl of Phe193^ECL2^. This conformation can be described as “intracellular opening coupled to extracellular closure.” While the intracellular surface opens to accommodate the G protein, the extracellular entrance and exit pathways for ligands become restricted. Consequently, both ligand association and dissociation rates may decrease [9]. Such observations provide a mechanistic explanation for how G protein coupling enhances agonist affinity (Figure 4).

Importantly, the degree of pocket closure and the network of local interactions are influenced by the chemical nature and efficacy of the bound ligand. Full agonists, partial agonists, antagonists, and inverse agonists engage the orthosteric pocket through distinct interaction patterns and may differentially affect local conformations and interaction networks involving ECL2, TM5, TM6, and TM7. Therefore, the closed active state of β2AR should be regarded not as a single rigid structure, but as a dynamic ensemble of related active conformations stabilized by G protein coupling [43].

#### 4.2.2. M2 Muscarinic Acetylcholine Receptor (M2R)

The M2R provides an example of receptor activation that involves substantial remodeling of the ligand-binding pocket through a mechanism distinct from that observed in β2AR. In the active-state structure of M2R stabilized by the agonist iperoxo and a G protein-mimetic nanobody, M2R activation involves not only the expected conformational rearrangements on the cytoplasmic side but also pronounced structural changes in the extracellular region and the orthosteric binding site. In active M2R, the outward displacement of TM6 on the cytoplasmic side creates a conformation suitable for G protein binding. On the extracellular side, meanwhile, the upper portions of TM5, TM6, and TM7, together with the ECL region, are repositioned toward the ligand, resulting in marked contraction of the orthosteric binding site. This contraction has been reported to exceed the corresponding changes observed in the active states of β2AR and rhodopsin, suggesting that active M2R forms a pocket that more deeply encloses the agonist [44].

Thus, M2R illustrates how stabilization of the active state by either a G protein or a G protein-mimetic nanobody can be coupled to extensive remodeling of the extracellular ligand-binding region. Although both M2R and β2AR can be described in terms of extracellular closure, the structural elements involved and the mechanisms by which closure occurs differ substantially. M2R therefore extends the concept of the closed active state while simultaneously highlighting receptor-specific structural diversity.

#### 4.2.3. μ-Opioid Receptor (μOR)

Analgesia and euphoria mediated by opioids are primarily produced through μOR-dependent activation of Gi proteins. NMR analysis of μOR activation showed that agonist binding alone is insufficient to fully stabilize the receptor conformation required for G protein engagement; instead, conformational changes in TM5 and TM6 are markedly promoted when the agonist BU72 is combined with a G protein-mimetic nanobody. This suggests that intracellular transducer engagement strengthens the allosteric coupling between the agonist-binding pocket and the G protein coupling interface [45]. In agreement with this view, the cryo-EM structure of the agonist DAMGO-bound μOR–Gi complex revealed outward displacement of TM6 and formation of an intracellular cavity that accommodates the Gαi α5 helix, while DAMGO is stably retained within the orthosteric pocket. Although these studies do not directly demonstrate Gi-induced remodeling of the same ligand-binding pocket, they support the idea that Gi coupling stabilizes the active conformation of μOR and may allosterically influence the structure and dynamics of the ligand-binding pocket. In contrast to β2AR, where G protein coupling is associated with a clear closure of the extracellular ligand-binding pocket, the structural effect in μOR may be better understood as coordinated stabilization of the receptor core and ligand-binding mode in the active state [46].

#### 4.2.4. Adenosine A2A Receptor (A2AR)

A2A receptor signaling functions as a Gs-coupled counter-regulatory pathway that opposes Gi-coupled receptor signaling, particularly dopamine D2 receptor signaling. The A2AR provides an important counterexample demonstrating that G protein coupling does not necessarily induce large static rearrangements of the extracellular ligand-binding pocket. Structures of the agonist NECA-bound A2AR–mini-Gs complex revealed that the transition from an agonist-bound active-intermediate state to a G protein-bound active state is characterized primarily by conformational changes on the intracellular side of the receptor. These include an approximately 14 Å outward displacement of the cytoplasmic end of TM6, modest rearrangements of TM5 and TM7, and rotameric changes in conserved activation motifs.

In contrast, little structural change is observed in the extracellular half of the receptor surrounding the ligand-binding pocket. Thus, rather than inducing a pronounced extracellular pocket closure as proposed for β2AR, mini-Gs appears to stabilize the fully active intracellular conformation without substantially altering the agonist-bound ligand-binding site. A2AR therefore illustrates that reverse allostery from G proteins may not always manifest as a large structural rearrangement. Even when the average structure of the orthosteric pocket remains relatively unchanged, G protein coupling may influence ligand efficacy and coupling selectivity through effects on receptor dynamics, local fluctuations within the ligand-binding region, or shifts in conformational equilibria among active states [20]. Consequently, A2AR serves as an important reminder that the concept of a closed active state is not universally applicable across all GPCRs and may exhibit distinct structural and dynamic phenotypes depending on the receptor.

#### 4.2.5. β1-Adrenergic Receptor (β1AR)

Coupling of β1AR to Gs also plays a central role in stabilizing ligand-bound active conformations. However, unlike β2AR, β1AR does not exhibit a prominent lid-like closure of the extracellular pocket as a defining feature of activation. Instead, cryo-EM structures of β1AR–Gs complexes bound to ligands with different efficacies—including full agonists, partial agonists, and very weak partial agonists—have revealed largely similar overall active-state complex structures, accompanied by localized conformational differences primarily surrounding the ligand-binding pocket.

These local differences are associated with variations in complex stability and downstream signaling efficacy. Biochemical studies further showed that nucleotide-free β1AR–Gs intermediates formed in the presence of a full agonist are more stable than those formed with partial agonists. Importantly, molecular dynamics simulations comparing ligand-bound β1AR in the presence and absence of Gs indicate that Gs coupling reduces fluctuations not only at the intracellular side of the receptor but also in regions including the orthosteric ligand-binding pocket, consistent with allosteric stabilization of agonist binding by G proteins. Thus, β1AR provides an example in which communication between the orthosteric pocket and the Gs-coupling interface is manifested primarily through ligand-dependent local dynamics and complex stability, rather than through a pronounced extracellular pocket closure as proposed for β2AR [47].

Together, these examples indicate that G protein-mediated reverse allostery is not expressed as a uniform structural phenotype. Depending on the receptor, transducer coupling may promote extracellular pocket closure, orthosteric-site contraction, stabilization of an active binding mode, or more subtle dynamic changes without large static rearrangement. Thus, the closed active state should be considered one mechanistic manifestation of a broader principle: ligand recognition is shaped by the receptor–ligand–transducer ensemble (Table 2).

These examples indicate that G protein-mediated reverse allostery is not a uniform structural phenotype across class A GPCRs. Rather, it represents a general allosteric principle in which intracellular transducer engagement can reshape ligand recognition, while the structural manifestation of this coupling is highly receptor-dependent.

## 5. Pathophysiological and Drug Discovery Implications of G Protein-Mediated Regulation of Ligand Binding

The receptor-specific effects of G protein coupling on ligand binding suggest that the intracellular receptor–G protein interface plays an important role in the allosteric regulation of ligand recognition. Alterations at this interface may result from disease-associated mutations or pharmacological modulation. Consequently, changes in ligand recognition may occur that cannot be predicted solely from the architecture of the orthosteric binding pocket.

### 5.1. Craniofacial Malformations Caused by an ETAR Mutation and Allosteric Regulation of Ligand Affinity

**Figure 5:**
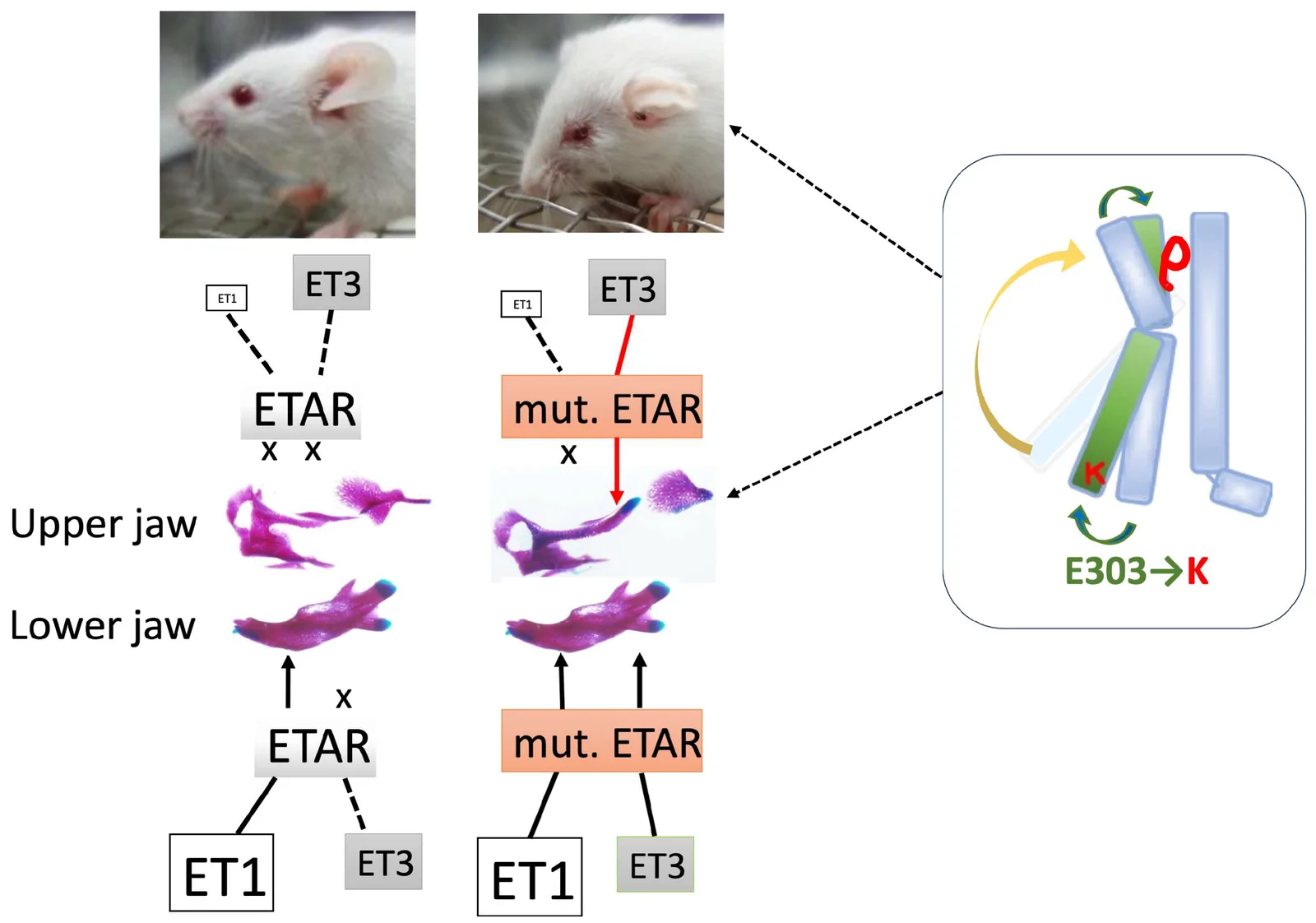
Proposed mechanism underlying the maxillary phenotype caused by mutant ETAR. In the normal upper jaw, ETAR signaling is absent or minimal because ETAR does not efficiently respond to ET3. In contrast, mutant ETAR acquires increased responsiveness to ET3, leading to ectopic signaling and mandibular-like bone, including articular cartilage formation in the maxillary region. Purple and blue staining indicate bone and cartilage, respectively. Arrows indicate ectopic mandibular-like bone formation, and X indicates absent or minimal ETAR signaling in the normal maxillary region.

In 2023, we identified the E303^6.32^K mutation in the endothelin A receptor (ETAR/EDNRA), a class A GPCR, as a recurrent mutation responsible for mandibulofacial dysostosis with alopecia (MFDA). Patients carrying the E303K mutation exhibited phenotypes characterized by malformations of craniofacial bones and middle-ear ossicles, partial eyelid defects, and alopecia. Mouse models harboring the same mutation also showed partial maxillary-to-mandibular transformation, together with abnormalities of the middle-ear ossicles and eyelids. These phenotypes in mice were completely rescued by genetic reduction or deletion of endothelin 3 (ET3/EDN3). Pharmacological analyses further demonstrated that the E303K mutant ETAR showed increased affinity for ET3 and enhanced ability to activate G proteins. Under physiological conditions, ETAR preferentially recognizes ET1 and ET2 and shows little responsiveness to ET3. In contrast, the E303K mutation increases ET3 affinity and responsiveness, indicating that E303K acts as an ET3-dependent gain-of-function mutation (Figure 5).

The importance of this study lies in the fact that the mutation enhances the affinity of ETAR for ET3 despite being located not within the ligand-binding pocket but at the intracellular end of TM6, away from the ligand-binding site. Although the crystal structure of ETBR has been determined, that of ETAR has not yet been resolved. Therefore, MD simulations were used to analyze the structural properties caused by the mutation. E303^6.32^K replaces a negatively charged glutamate with a positively charged lysine. This substitution was shown to increase the flexibility of the intracellular end of TM6 and to increase the probability that TM6 undergoes a large outward displacement, thereby facilitating receptor conformational states suitable for G protein binding. Consistent with this intracellular rearrangement, MD simulations showed that the extracellular ligand-binding pocket more frequently adopted a contracted conformation, thereby providing a structural basis for the increased affinity of the mutant receptor for ET3 [10].

Thus, the E303K mutant ETAR can be regarded as an important example showing, in an in vivo disease model, that intracellular conformational changes associated with G protein binding can influence the extracellular ligand-binding pocket through the entire receptor, leading to contraction of the pocket. As a result, ETAR signaling becomes enhanced in regions where it would not normally be activated during development, leading to the craniofacial malformations observed in MFDA.

In addition to E303^6.32^K, the Y129^2.53^F mutation also produces similar craniofacial phenotypes in both humans and mice and acts as an ET3-dependent gain-of-function mutation by increasing the affinity of ETAR for ET3. However, the structural basis of this effect differs from that of E303^6.32^K. Whereas E303^6.32^K is located at the intracellular end of TM6 and promotes conformational states favorable for G protein binding, Y129^2.53^ is located beneath the ligand-binding pocket, above the sodium–water pocket. Replacement of this residue with phenylalanine reduces water retention within the sodium–water pocket and weakens the sodium–water network, thereby increasing the probability that the ligand-binding pocket adopts a contracted conformation favorable for ET3 binding [10]. Together, these findings show that spatially distinct ETAR mutations can enhance ET3 affinity through different allosteric mechanisms: E303K through intracellular conformational changes linked to G protein coupling, and Y129F through remodeling of the sodium–water pocket beneath the ligand-binding site.

ETAR therefore represents a particularly direct disease-associated example in which distal mutations alter ligand recognition through long-range allosteric communication. However, clinical examples in which mutations at the G protein coupling interface have been clearly shown to modify ligand binding through G protein-mediated reverse allostery remain limited. Nevertheless, in vitro mutational studies support the broader concept that intracellular regions and activation motifs of GPCRs can allosterically influence ligand binding. For example, a constitutively active β2-adrenergic receptor mutant involving the third intracellular loop exhibits increased agonist affinity without a corresponding increase in antagonist affinity [5]. Similarly, in the histamine H4 receptor, mutation of the conserved R3.50 residue in the DRY motif reduces affinity for histamine while increasing affinity for the inverse agonist thioperamide [48]. These findings indicate that structural perturbations outside the orthosteric pocket can influence ligand binding by shifting receptor conformational equilibria. Although numerous disease-associated GPCR mutations have been identified, the pathogenic mechanisms of many such mutations remain incompletely understood. Therefore, some unexplained disease-associated mutations may involve altered ligand recognition mediated by intracellular activation motifs, G protein coupling regions, or other receptor-wide allosteric networks. Thus, ETAR should be regarded not as proof of a universal mechanism, but as a direct disease-associated example that underscores the need to analyze ligand recognition, receptor dynamics, and transducer coupling as interconnected features of mutant GPCR pharmacology.

### 5.2. Stabilization of Ternary Complexes by Positive Allosteric Modulators and Its Application to Drug Discovery

The stabilization of G protein-bound receptor complexes by positive allosteric modulators (PAMs) provides a useful framework for linking molecular pharmacology to therapeutic effects. A representative example is the A1 adenosine receptor (A1R), which has attracted attention as a potential target for non-opioid analgesic therapy. In the active A1R complex, the orthosteric agonist adenosine, the PAM MIPS521, and the Gi2 heterotrimer are simultaneously bound to the receptor. MIPS521 further stabilizes the resulting agonist-bound receptor–G protein complex. MIPS521 occupies an extrahelical allosteric pocket exposed to the lipid or detergent environment, outside the orthosteric binding site, and this pocket is formed by the outer surfaces of TM1, TM6, and TM7. Gaussian accelerated molecular dynamics (GaMD) simulations and ligand kinetic binding experiments further supported a model in which Gi2 binding stabilizes the active conformation of adenosine-bound A1R, and MIPS521 provides additional stabilization of the adenosine–A1R–Gi2 complex. Thus, MIPS521 appears to function not simply as an enhancer of orthosteric adenosine signaling, but as a PAM that facilitates the formation or persistence of the G protein-bound agonist–receptor complex [49].

The physiological relevance of this mechanism is particularly evident under pathological conditions. In neuropathic pain, endogenous adenosine levels are locally elevated in the spinal cord. MIPS521 exerted analgesic effects not by directly activating the receptor, but by stabilizing the adenosine–A1R–Gi complex formed by this endogenous adenosine. Therefore, this PAM can be regarded as a disease-dependent potentiator that acts by exploiting the increased endogenous adenosine tone present under pathological conditions. These findings provide an important example showing that stabilization of a G protein-bound ligand–receptor complex can be linked not only to the regulation of intracellular signaling, but also to pharmacological effects at the organismal level, namely analgesia [49].

A similar concept is also supported by studies of the M2 muscarinic acetylcholine receptor (M2R), a Gi/o-coupled inhibitory muscarinic receptor. In an experimental system in which purified M2R was reconstituted into nanodiscs, G protein coupling promoted formation of the agonist-bound high-affinity state, after which a PAM further stabilized the agonist–receptor–G protein ternary complex. In the presence of nucleotides, stabilization of this ternary complex by the PAM led to an increase in the initial rate of signaling. These findings indicate that the orthosteric ligand, the G protein, and the allosteric modulator can cooperatively stabilize the high-affinity active state of a GPCR. In this sense, the G protein can be regarded as an endogenous allosteric stabilizer of the agonist-bound active state, whereas an exogenous allosteric modulator may act from the extracellular side or the membrane-facing region, allowing both factors to cooperatively regulate the same active receptor state [50]. Such observations underscore the importance of drug design strategies that target not only the orthosteric binding site, but also the stability of the G protein-bound active state and the ternary complex in GPCR drug discovery.

The pathophysiological and drug-discovery implications discussed above are summarized in Table 3.

ETAR mutations show that distal allosteric perturbations can alter ligand selectivity in disease, whereas A1R/MIPS521 and M2R/PAM studies demonstrate that stabilization of ligand–receptor–G protein complexes can be exploited for therapeutic modulation.

## 6. Conclusions and Future Perspectives for Drug Discovery

The evidence reviewed here indicates that GPCR pharmacology cannot be understood solely from the orthosteric ligand-binding pocket. Ligand binding, receptor activation, G protein coupling, nucleotide state, and allosteric modulation are integrated through receptor-wide allosteric networks that shape conformational ensembles [1,4,6,7,42,51,52]. This ensemble-based view is particularly important for GPCR drug discovery, because therapeutically useful ligands or modulators may act by stabilizing selected receptor–ligand–transducer states rather than simply by binding to a static pocket. This reverse allosteric view has direct implications for pharmacology: by transmitting the effects of G protein binding through the transmembrane helical bundle to the extracellular ligand-binding pocket, receptor–G protein complexes can alter receptor pharmacology and ligand recognition, and even ligand selectivity [9,10,53,54]. Such reverse allostery provides a molecular basis for the classical high-affinity agonist-binding state and redefines drug action as a state-dependent phenomenon of the receptor–G protein complex [4,7,9]. Importantly, G protein-mediated reverse allostery should not be equated with a universal closed-pocket mechanism; depending on the receptor, it may manifest as extracellular closure, pocket contraction, active-state stabilization, or altered local dynamics without large static rearrangement.

Findings from several receptor systems suggest that G protein-mediated modulation of the ligand-binding pocket may represent a broadly relevant regulatory mechanism in class A GPCRs. Nevertheless, the molecular consequences of such modulation cannot be fully predicted from static receptor structures alone. How dynamic redistribution among transient pocket conformations shapes ligand association and dissociation kinetics remains incompletely understood. In addition, factors such as the nucleotide state of bound G proteins [7,18,28,29], membrane phosphoinositides and receptor phosphorylation patterns [55,56,57], and receptor dynamics captured by molecular simulations [58] may further reshape receptor–transducer conformational ensembles. Integrating time-resolved structural methods with ligand kinetic measurements will therefore be essential for defining which conformational transitions are directly responsible for altered ligand recognition. Future screening strategies may need to evaluate ligand kinetics in defined receptor–transducer states, including nucleotide-free, nucleotide-bound, and partially coupled intermediates. Such approaches would move beyond equilibrium affinity measurements in receptor-only systems and may better capture state-dependent effects on ligand association, dissociation, and residence time [9,53,54].

PAMs should therefore be considered not only as enhancers of orthosteric ligand affinity, but also as stabilizers of therapeutically favorable ternary complexes and potential modulators of ligand-binding kinetics [49,50]. This mechanism may be especially advantageous when endogenous ligands are increased locally under pathological conditions, allowing signaling to be potentiated in a disease-context-dependent manner [49]. Such context-dependent modulation offers drug discovery advantages distinct from those of conventional agonists or antagonists, because it may allow signaling to be enhanced preferentially in relevant tissues or pathological environments while avoiding systemic receptor activation. Thus, it will be important to consider PAMs not only as “affinity enhancers,” but also as “ternary complex stabilizers” or “kinetic modulators” [49,50].

Disease-associated GPCR mutations further illustrate the need for an ensemble-based perspective. The E303K and Y129F mutations in ETAR show that perturbations distant from the orthosteric pocket can reorganize ligand selectivity through long-range allostery, indicating that pathological GPCR activation involves altered ligand recognition as well as altered signaling efficacy [10]. In the future, drug design strategies that target conformational ensembles specific to mutant receptors and correct abnormal ligand binding, excessive G protein coupling, or pathological active states may prove useful [42,51].

To realize such drug discovery approaches, these time-resolved and kinetic analyses should be combined with NMR [59], EPR/DEER [60], molecular dynamics simulations [58], and coupling databases or structure-prediction resources [61,62] to define drug-stabilized receptor states more precisely. GPCR–G protein complexes do not exist as single stable structures, but dynamically transition among multiple intermediate states, pre-coupled states, and fully active states [7,28,29,39,42]. Therefore, understanding which states are stabilized by a drug, and which transitions are promoted or suppressed, will provide a basis for improving receptor subtype selectivity, G protein selectivity, signaling bias, and tissue selectivity.

In summary, allosteric regulation of ligand binding by G proteins provides a conceptual framework that connects classical pharmacological models, recent structural biology, the molecular mechanisms of disease-associated mutations, and next-generation GPCR drug discovery. Future GPCR drug discovery should not focus solely on the orthosteric binding pocket but should also target the dynamic allosteric network of the entire receptor that is formed upon G protein binding. This perspective may facilitate the design of drugs with improved receptor selectivity, pathway selectivity, kinetic control, and disease-context-dependent actions.

## Figures and Tables

**Table 2:** Summary of representative GPCR systems showing G protein–dependent modulation of ligand binding and receptor pharmacology.

Receptor	G Protein	Main Evidence	Effect on Ligand-Binding Pocket	Key Implication	Ref.
β2AR	Gs	biochemical/structural	closed active state, restricted ligand access	residence time, high-affinity state	[9,43]
M2R	Gi/o mimic	active-state structure	orthosteric-site contraction	receptor-specific closure mechanism	[44]
μOR	Gi	NMR/ cryo-EM	active-state stabilization	coupling-linked ligand mode	[45,46]
A2AR	mini-Gs	crystal structure	little static extracellular rearrangement	dynamic rather than structural reverse allostery	[20]
β1AR	Gs	cryo-EM/MD	local ligand-pocket flexibility	efficacy-dependent complex stability	[47]
ETAR	Gq-related context	disease mutation/ in vivo/MD	pocket contraction via distal mutations	pathological ligand selectivity	[10]

**Table 3:** Pathophysiological and drug-discovery implications of allosteric regulation of ligand binding.

Context	System	Key Mechanism	Main Implication	Ref.
Disease-associated ligand selectivity	ETAR E303K	Intracellular TM6 mutation promotes ET3-dependent gain of function and ligand-pocket contraction	A distal intracellular mutation can alter ligand selectivity and cause developmental disease.	[10]
Disease-associated ligand selectivity	ETAR Y129F	sodium–water network remodeling increases ET3 affinity	Distinct allosteric routes produce similar pathological ligand selectivity and cause developmental disease.	[10]
Disease-context- dependent PAM action	A1R/ MIPS521	MIPS521 stabilizes the adenosine–A1R–Gi complex under elevated endogenous adenosine conditions	PAMs can potentiate endogenous signaling in disease contexts.	[49]
Ternary-complex stabilization	M2R/PAM	PAM stabilizes the agonist–M2R–G protein ternary complex after G protein-promoted high-affinity-state formation	Drug design can target ternary- complex stability, not only orthosteric affinity.	[50]

## Data Availability

No new data were created or analyzed in this study. Data sharing is not applicable to this article.

## Abbreviations

The following abbreviations are used in this manuscript:

A1R	A1 Adenosine receptor
A2AR	Adenosine A2A receptor
AHD	α-helical domain
BRET	Bioluminescence resonance energy transfer
Cryo-EM	Cryo-electron microscopy
CTC	Cubic ternary complex
DEER	Double electron–electron resonance
ECL	Extracellular loop
ET	Endothelin
ETAR	Endothelin A receptor
ETC	Extended ternary complex
FRET	Fluorescence resonance energy transfer
GaMD	Gaussian accelerated molecular dynamics
GPCR	G protein-coupled receptor
HDX-MS	Hydrogen–deuterium exchange mass spectrometry
HRF-MS	Hydroxyl radical footprinting mass spectrometry
ICL	Intracellular loop
M2R	M2 Muscarinic acetylcholine receptor
NMR	Nuclear magnetic resonance
PAM	Positive allosteric modulator
TC	Ternary complex
TM	Transmembrane
β1AR	β1-Adrenergic receptor
β2AR	β2-adrenergic receptor
μOR	μ-Opioid receptor
